# Langerhans cell histiocytosis: unusual bone marrow infiltration—report of 2 cases in Ecuador

**DOI:** 10.3389/fmed.2024.1433463

**Published:** 2024-07-16

**Authors:** Paulina Santana, Marlon Arias-Intriago, Juan S. Izquierdo-Condoy

**Affiliations:** ^1^Department of Pathology, Medical Science Faculty, Universidad Central del Ecuador, Quito, Ecuador; ^2^Department of Anatomic Pathology, Hospital de Especialidades Carlos Andrade Marín, Quito, Ecuador; ^3^One Health Research Group, Faculty of Medicine, Universidad de las Américas, Quito, Ecuador

**Keywords:** bone marrow, histiocytes, Langerhans cells, Latin America, Ecuador

## Abstract

Langerhans cell histiocytosis (LCH) is a histiocytic neoplasm characterized by the abnormal proliferation of Langerhans cells. Bone marrow (BM) involvement is associated with high-risk disease and poor survival. Although BM involvement is particularly uncommon, no reported cases of LCH with BM infiltration have been documented in Latin America until now. The aim of this report is to highlight the clinical, hematological, and BM findings of two cases of LCH with BM infiltration, providing insights that may aid in detecting suspected patients. We present two cases of LCH with BM infiltration. One case involved a 23-month-old male patient, and the other a 16-month-old female patient. Common clinical findings in both cases included hepatosplenomegaly and fever. Hematological findings revealed anemia in both cases. The key diagnostic tool was the BM biopsy, which revealed histiocyte nests with characteristic morphology, CD1a-positive cells, increased eosinophils, and reactive paratrabecular lymphocytes. This report underscores the significance of clinical profiles in predicting BM infiltration in LCH. The presence of histiocyte nests displaying the characteristic morphology of Langerhans cells, accompanied by an elevation in eosinophils, indicates bone marrow involvement. Furthermore, the demonstration of CD1a-positive cells through immunohistochemistry serves as a crucial diagnostic tool.

## 1 Introduction

Langerhans cell histiocytosis (LCH) is a histiocytic neoplasm characterized by the abnormal proliferation of Langerhans cells driven by sporadic activating mutations in the MAPK pathway (1–3). It can manifest at any age, but more than 60% of cases occur before the age of 2, with an incidence of 1 to 2 newborns per million per year (4, 5).

High-risk LCH is defined as multisystemic infiltration involving high-risk organs like bone marrow (BM). Identifying Langerhans cells in the BM is rare, and suspicion should be raised and evaluated in LCH patients presenting with single or multiple cytopenias on peripheral blood assessment (3, 4, 6). BM trephine biopsy is utilized to demonstrate, through immunohistochemistry, the presence of CD1a-positive Langerhans cells (3).

Tissues such as bones and skin are the most commonly affected by LCH, while bone marrow (BM) involvement represents one of the less common targets, with limited cases reported to date. In this context, it appears that no cases of LCH with bone marrow infiltration have been reported in Ecuador or the region.

We present two pediatric cases with Langerhans cell histiocytosis involving the bone marrow.

## 2 Case presentation

A retrospective analysis of bone marrow examination reports from the Anatomical Pathology Department was conducted between January and June 2023.

### 2.1 Case 1

A 23-month-old male, with no reported family history and no other known comorbidities, was brought by his mother due to a fever quantified at 38°C and chills 10 days earlier. He was initially sent home with paracetamol and cetirizine for a suspected upper respiratory viral infection. However, the patient returned due to a poor clinical response, plus 48 h of polyarthralgia and lower limb edema.

Clinical findings upon return revealed bilateral diffuse lung crackles, a fever of 38°C, and a body mass index of 16.3 kg/m^2^. Blood analysis showed a hemoglobin (Hb) level of 6.7 g/dL (reference range 12–16 g/dL), a Total Leukocyte Count (TLC) of 3,573/μL (reference interval 4–10^3^/μL), and platelets of 21,000/μL (reference range 130,000–400,000/μL; Table 1). A chest X-ray displayed bilateral diffuse infiltrates. The patient was admitted with a diagnosis of pancytopenia, neutropenic fever, and pneumonia. Hemocultures and urocultures were taken, a two-unit blood pack was administered, and antibiotic therapy with cefepime, vancomycin, and trimethoprim-sulfamethoxazole was initiated.

**Table 1:** Clinic-hematological profile, BM findings and tumoral cells immunophenotype of LCH patients with BM infiltration.

Patients’ characteristics	Case1	Case2
Age at diagnosis (months)	23	16
Sex	Male	Female
Fever	+	+
Skin rash	−	−
Hepatomegaly	+	+
Splenomegaly	+	+
Lymphadenopathy	−	+ (iliac, intercaval-aortic, cervical)
Bony lesion	−	−
Soft tissue lesion	−	−
Another site/CNS /Lung		Pneumonia, pleural effusion.
**Hematological characteristics**		
Hb (gm/dl)	6.7	8.6
TLC (^3^/μL)	3.57 × 109	15.4 × 109
Platelets (/μL)	21 × 109	306 × 109
**Post-chemotherapy hematological characteristics**		
Hb (gm/dl)	11.7	10.1
TLC (^3^/μL)	4.1 × 109	4.3 × 109
Neutrophils (/μL)	2,750	680
Platelets (/μL)	241 × 109	303 × 109
**Bone marrow characteristics**		
**Cellularity**	**Hypocellular (60%)**	**Normocellular (100%)**
Histiocytes	Diffuse	Patchy
Hemophagocytes	−	+ (in aspirate)
Eosinophils	+	+
Fibrosis	Diffuse	Patchy
Cellular background in all intertabercular spaces	Atypic lymphoid aggregates, and atypic small megakaryocytes with hypolubulated, hyperchromatic nucleus.	Erythroid, Myeloid, Megakaryocyte (normal)
Necrosis	−	−
Plasma cells	+ polyclonal	+
Megakaryocytes	1/field dysplastic	1/field normal
M:E relationship	1:1	3:1
CD1a	+ 100/field	+ >100/field
**Tumoral cells immunophenotype**		
CD1a	+ 100/field	+ >100/field
S100	+	+
CD68	+	+
Cyclin D1	+	

**Figure 1:**
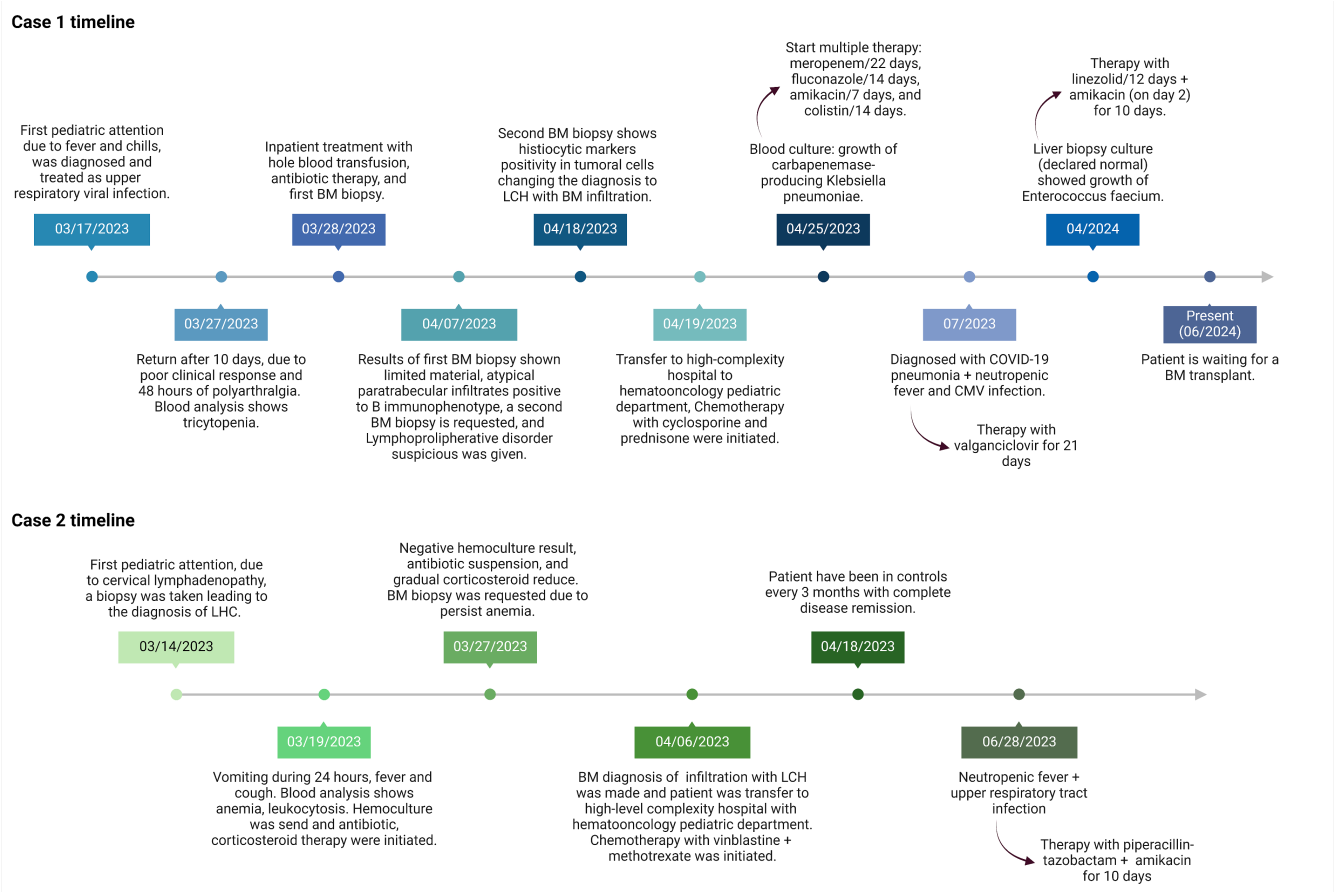
Timeline of patients 1 and 2 with LCH.

A BM biopsy was performed due to pancytopenia, revealing a limited sample with two intertrabecular spaces, small, atypical megakaryocytes, and atypical paratrabecular lymphoid infiltration with the predominance of a B mature immunophenotype (Supplementary Figure 1), initially suggesting a lymphoproliferative disorder with BM infiltration. Due to this diagnosis, the patient was referred to a high-level pediatric oncology hospital for another BM biopsy, as the initial sample had limited material (Figure 1).

**Figure 2:**
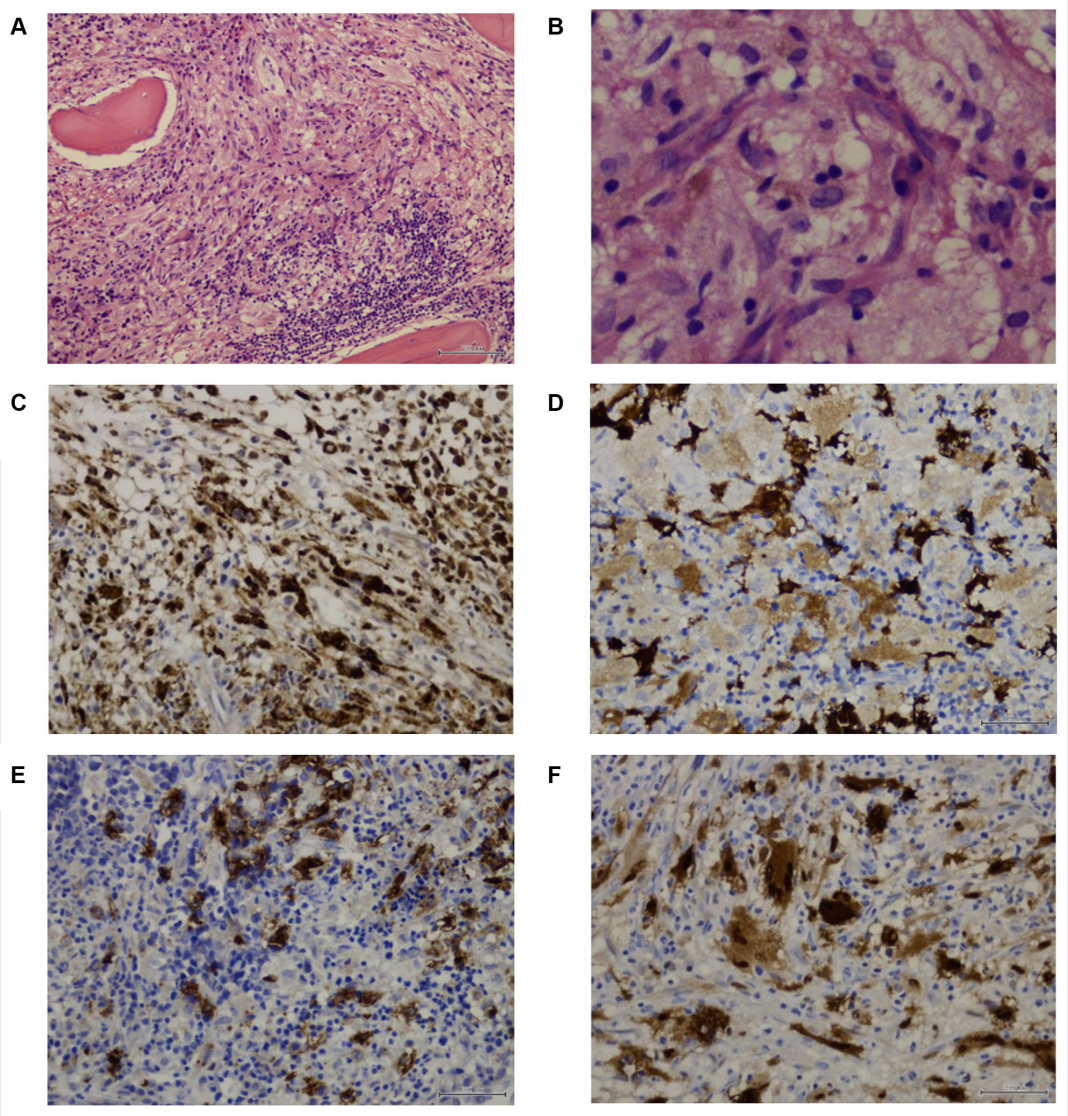
Patient 1 subsequent bone marrow biopsy. Panel of microphotographs from bone marrow trephine biopsy of a case 1 of LCH indicating BM infiltration by Langerhans Cells: **(A)** Bone marrow trephine biopsy showing a diffuse area comprising of Langerhans cells with a multinucleated giant cell and some eosinophils (Hematoxylin and Eosin stain, magnification 100X). **(B)** Bone marrow trephine biopsy showing the morphology of Langerhans cells at higher magnification, the cells having grooved nuclei (Hematoxylin and Eosin stain, magnification 400X). **(C)** Immunohistochemistry for CD68 showing membranous positivity in histiocytes and Langerhans cells (magnification 200X). **(D)** Immunohistochemistry for S100 showing nuclear positivity in Langerhans cells (magnification 200X). **(E)** Immunohistochemistry for CD1a showing membranous positivity in Langerhans cells (magnification 200X). **(F)** Immunohistochemistry for Cyclin D1 showing nuclear positivity in Langerhans cells, and multinucleated Histiocyte (magnification 200X).

A subsequent BM core biopsy was examined 2 weeks later, showing an adequate BM biopsy with diffuse multiple atypical multinucleated cells (Figures 2A,B). An extensive immunophenotype panel was requested, where CD1a, CD68, Cyclin D1, and S100 were positive in atypical multinucleated cells (Figures 2C–F). This confirmed the diagnosis of BM infiltration with LCH and concluded that the atypical paratrabecular lymphoid aggregates were reactive. While the patient’s pulmonary condition improved, reducing the crackles, pancytopenia persisted. After 18 days, he was transferred to the pediatric hematology-oncology department in a high-complexity hospital, where he received chemotherapy with cyclosporine and prednisone for 4 weeks. Treatment response showed an increase in hemoglobin and platelets, but neutropenic fever persisted.

The 9:22 translocation was assessed using PCR and FISH, with negative results. Flow cytometry showed a normal CD4/CD8 ratio (1.7), a normal SLGKAPPA+/SLGLAMBDA+ ratio (1.2), and no aberrant expansions. The patient undergoes monthly check-ups; an abdominal ultrasound in April 2023 showed a liver size of 9.7 cm (normal range 8.3–10.5 cm) and a spleen size of 9.6 cm (normal range < 8.3 cm). The first chemotherapy cycle was conducted in May 2023. By June 2023, the liver size had increased to 10.7 cm and the spleen size to 10.6 cm. A post-chemotherapy bone marrow biopsy conducted in June 2023 showed an increase in hematopoietic cells compared to the previous biopsy, with 10% of bone marrow involvement by CD1a + histiocytes, and it remained hypocellular. Complete blood counts have fluctuated over time, necessitating multiple blood transfusions and platelet concentrates, totaling 44 in all. As of June 2024, 12 months after the initial diagnosis, he is a candidate for a bone marrow transplant and is awaiting the procedure.

### 2.2 Case 2

A 16-month-old female, with no relevant family history and no other known comorbidities, was brought by her mother due to continuous vomiting for 24 h, fever, and cough. She was admitted with a diagnosis of anemia, fever, and cervical lymphadenopathy. Clinical examination showed a temperature of 38.5°C, hepatosplenomegaly 8 cm below the costal margin, and multiple tender bilateral inguinal and cervical lymphadenopathy less than 1 cm in size. Blood analysis revealed a hemoglobin (Hb) level of 8.6 g/dL (reference range 12–16 g/dL), a Total Leukocyte Count (TLC) of 15,433/μL (reference interval 4–10^3^/μL), and platelets of 430,000/μL (reference range 130,000–400,000/μL; Table 1).

**Figure 3:**
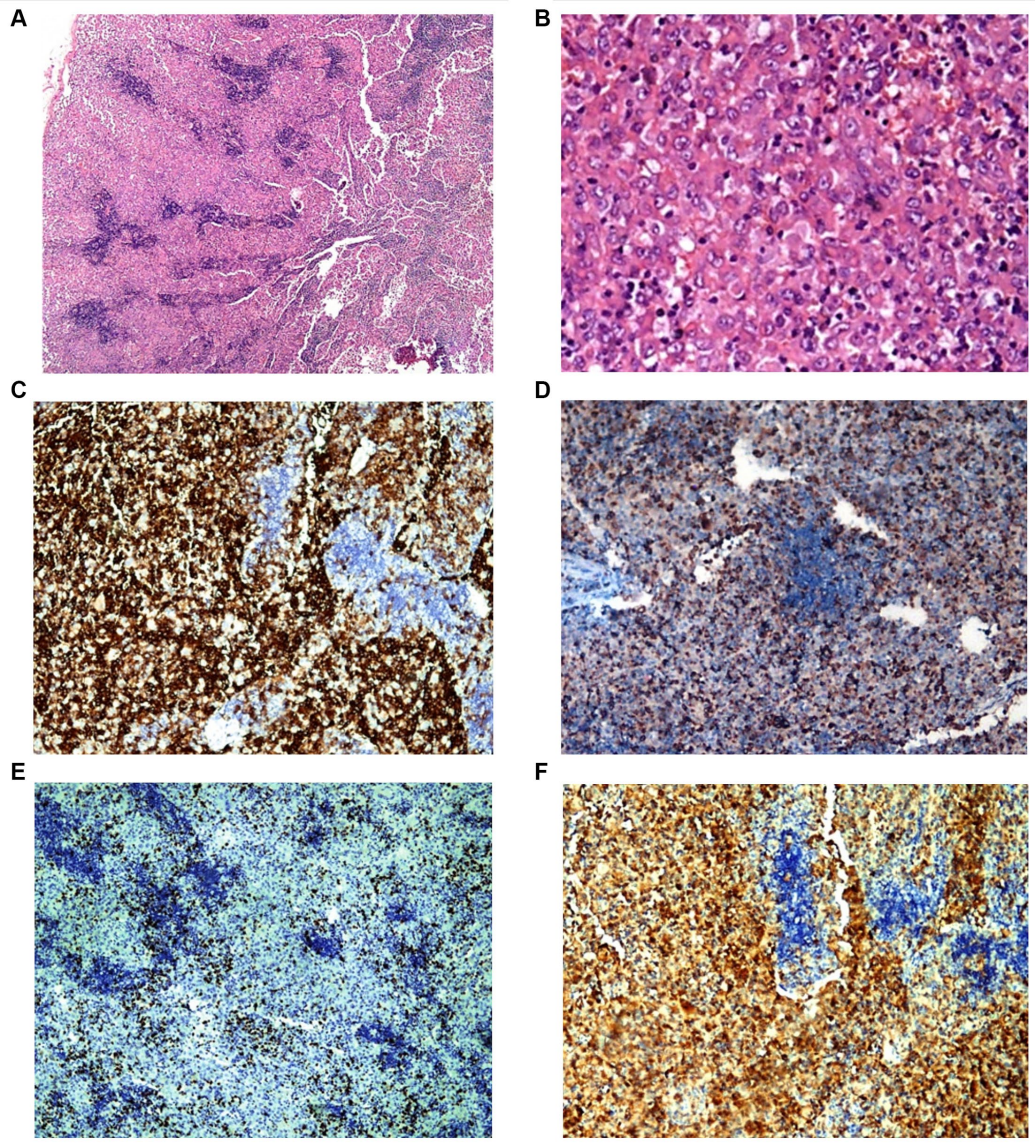
Patient 2 lymph node biopsy. Panel of microphotographs from cervical lymph node biopsy of patient 2 indicating LCH: **(A)** Lymph node biopsy showing a patchy infiltration of Langerhans cells (Hematoxylin and Eosin stain, magnification 40X). **(B)** Lymph node biopsy showing the morphology of Langerhans cells at higher magnification (Hematoxylin and Eosin stain, magnification 400X). **(C)** Immunohistochemistry for CD1a showing membranous positivity in Langerhans cells (magnification 100X). **(D)** Immunohistochemistry for CD68 showing membranous positivity in histiocytes and Langerhans cells (magnification 100X). **(E)** Immunohistochemistry for KI67 showing nuclear positivity in Langerhans cells with 10–15% KI67 index (magnification 100X). **(F)** Immunohistochemistry for S100 showing nuclear positivity in Langerhans cells (magnification 100X).

**Figure 4:**
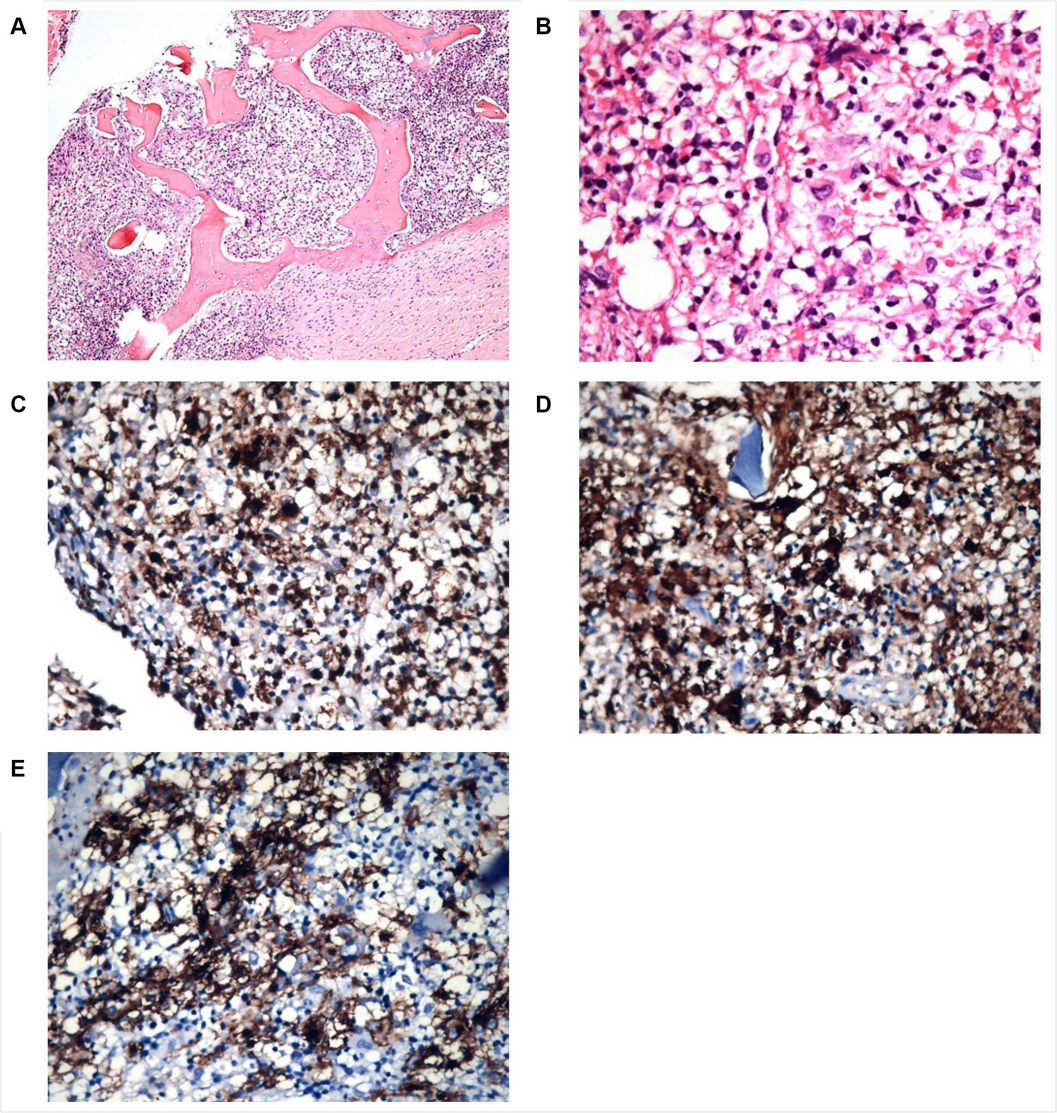
Patient 2 bone marrow biopsy. Panel of microphotographs from bone marrow trephine biopsy of patient 2 of LCH indicating BM infiltration by Langerhans Cells: **(A)** Bone marrow trephine biopsy showing a patchy infiltration of Langerhans cells and some eosinophils (Hematoxylin and Eosin stain, magnification 100X). **(B)** Bone marrow trephine biopsy showing the morphology of Langerhans cells at higher magnification, the cells having grooved nuclei (Hematoxylin and Eosin stain, magnification 400X). **(C)** Immunohistochemistry for CD68 showing membranous positivity in Langerhans cells (magnification 100X). **(D)** Immunohistochemistry for S100 showing nuclear positivity in Langerhans cells (magnification 100X). **(E)** Immunohistochemistry for CD1a showing membranous positivity in Langerhans cells (magnification 100X).

A cervical lymph node biopsy was performed, revealing interstitial infiltrations of histiocytes positive for CD1a, CD68, and S100, leading to the diagnosis of LCH (Figure 3). Due to the suspicion of bone marrow (BM) infiltration associated with moderate anemia, a BM trephine biopsy was requested, confirming patchy interstitial infiltrations of histiocytes with CD1a, CD68, and S100 positivity in the tumor cells (Figure 4). A transfer to a Pediatric Hematology-Oncology unit in a high-complexity hospital was requested.

Parenteral hydration, prednisone, and empiric antibiotic therapy with ceftazidime were initiated after a hemoculture was taken. After 5 days, the hemoculture showed no bacterial growth, leading to antibiotic suspension and a reduction in prednisone dosage, although fever peaks persisted. After 6 days, the patient was transferred to a high-complexity hospital, where chemotherapy with vinblastine and methotrexate was initiated for 6 weeks. This led to a positive clinical response, with cessation of fever and an increase in Hb (Figure 1). Post-chemotherapy, the complete blood count remained stable, with three episodes of anemic crises requiring blood transfusions over time and one antibiotic scheme due to neutropenic fever.

As of 2024, 12 months after the initial diagnosis, the patient has been attending regular check-ups every 3 months and has achieved complete remission, with no lymphadenopathy or anemia.

## 3 Discussion

LCH is a rare histiocytic neoplasm that can occur at any age, but more than 60% of cases occur before the age of 2, with an incidence of 1 to 2 newborns per million per year (3, 4, 7). The Hispanic race/ethnicity and low socioeconomic conditions directly affect LCH development. A SEER study of 145 cases indicated that Hispanics experience higher age-standardized LCH incidence rates than non-Hispanics (RR = 1.63, 95% CI: 1.15–2.29, *p* < 0.05). A study at Children’s Hospital Los Angeles reported a higher proportion of Hispanic cases compared to white cases in their study population (53% vs. 18%, respectively) (8).

Bone and skin are the most commonly involved organs in LCH. Involvement of the BM is uncommon, with a few reported incidences to date (5). Kumar M. et al. reported BM involvement in 26.3% (5 cases) out of 19 diagnosed cases of LCH. Another study showed approximately 33.3% BM involvement; however, larger studies reported relatively fewer cases, ranging from 2% to 7.5% (1). Nationally, in Ecuador, there is no precise data describing the status of LCH. However, records from the largest cancer hospital in Quito reported 40 cases of LCH diagnosed between 2011 and 2015, with an equal distribution between men and women (20 cases each) and no records of BM infiltration (9). While this report could suggest that BM involvement in LCH is not uncommon, the two cases in this report were the first with BM infiltration in our institution. The unexpected clustering of cases in a short period of time could be explained by the complexity of our hospital, which receives cases from almost all over the country.

Most patients with BM involvement are young children under the age of 2, as in our report, presenting with diffuse disease in the liver, spleen, lymph nodes, and skin, along with significant thrombocytopenia (<100,000/μL) and anemia (hemoglobin < 10 g/dL; infants, <9 g/dL), not secondary to other causes, and sometimes accompanied by leucopenia (<4^3^/μL) (2, 5, 10).

In one of our cases, we found lymphocyte aggregates in a paratrabecular arrangement and small, atypically hypolobulated, hyperchromatic nuclei in megakaryocytes. These findings raised suspicion of an underlying pathology, prompting a second bone marrow biopsy that led to the diagnosis of LCH. Besides these common findings, we found eosinophils in the BM infiltration background. The aspirate in Case 1 was deemed adequate, with calculated cellularity at 50% (hypocellular), an M:E ratio of 1:1, and the absence of megakaryocytes and platelets in the background.

In high-risk patients, LCH originates from CD34-positive stem cells with a BRAF *V600E* mutation in the BM, suggesting a myeloid dendritic cell origin (1, 4, 6).

We suggest, as Kumar M. et al. mentioned in his case report, that even in patients with LCH with single cytopenia, a BM aspirate and biopsy should be performed to assess BM infiltration (5). Staining the biopsy with anti-CD1a facilitates the detection of LCH cells. A pathognomonic feature of BM involvement is the presence of CD1a-positive tumor cells (2, 10–12). While we advocate for the inclusion of the Langerin marker, unfortunately, we were unable to conduct this test.

Regarding management, although some localized tumors can be treated surgically, the lymph node and bone marrow involvement that characterizes these patients usually requires systemic chemotherapy (13). The Histiocyte Society has unified criteria and established a treatment protocol based on etoposide, vinblastine, and prednisone (13). Some studies suggest that cyclosporine may be used as an alternative; however, its efficacy is limited (14). Due to the more aggressive clinical presentation and poor prognosis associated with bone marrow involvement, the patients in this report were treated with systemic chemotherapy (cyclosporine for the patient 1 and vinblastine + methotrexate for the patient 2) and steroids. Although the criteria for bone marrow transplantation are not fully established, it is indicated as a last resort after the failure of chemotherapy in these patients (15), a course of action proposed for patient 1 in this report.

This report emphasizes that the clinical profile of patients with LCH should be considered a crucial factor in predicting the occurrence of the disease. Bone marrow involvement is indicated by the presence of histiocyte nests displaying the characteristic morphology of Langerhans cells, accompanied by an elevation in eosinophils. Additionally, the demonstration of CD1a-positive cells through immunohistochemistry serves as a vital diagnostic tool. This case extends the diagnostic and histopathologic understanding within the Latin American population at risk for bone marrow infiltration in cases of LCH.

## 4 Patient perspective

Both patients and their families experienced significant stress and uncertainty due to the rare and challenging nature of Langerhans cell histiocytosis (LCH). Early diagnosis and comprehensive management, facilitated by a multidisciplinary team, provided clarity and direction for treatment. The journey highlighted the importance of specialized care, patient education, and regular follow-ups in ensuring optimal outcomes. Their progress underscores the need for continuous research and awareness of LCH to improve early detection and treatment strategies.

## Data availability statement

The original contributions presented in the study are included in the article/Supplementary material, further inquiries can be directed to the corresponding author.

## Ethics statement

Written informed consent was obtained from the individual(s) for the publication of any potentially identifiable images or data included in this article.

## Author contributions

PS: Conceptualization, Data curation, Formal analysis, Investigation, Methodology, Resources, Supervision, Validation, Writing – review & editing. MA-I: Conceptualization, Investigation, Methodology, Resources, Supervision, Validation, Writing – original draft. JI-C: Funding acquisition, Investigation, Methodology, Project administration, Resources, Supervision, Validation, Visualization, Writing – original draft, Writing – review & editing.

## Supplementary material

SUPPLEMENTARY FIGURE 1 Patient 1 first BM biopsy, showing atypical para trabecular lymphoid aggregates (Hematoxylin and Eosin stain, magnification 100X).
