# Supplementary figures and images for: Langerhans cell histiocytosis: unusual bone marrow infiltration—report of 2 cases in Ecuador
Source: Front Med (Lausanne). 2024 Jul 16;11:1433463. doi: 10.3389/fmed.2024.1433463 (PMC11286394; doi:10.3389/fmed.2024.1433463)

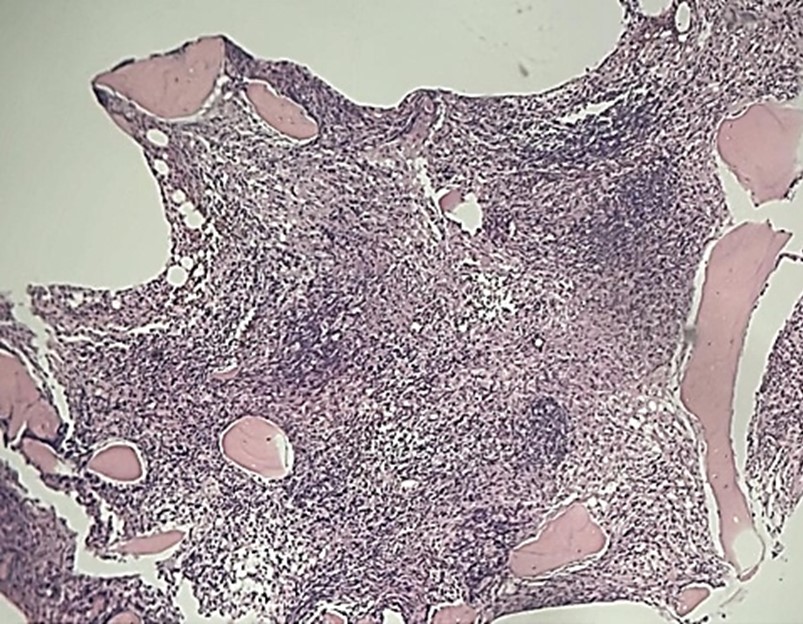

Supplement: SUPPLEMENTARY FIGURE 1 — Patient 1 first BM biopsy, showing atypical para trabecular lymphoid aggregates (Hematoxylin and Eosin stain, magnification 100X). [file Image_1.JPEG]
